# LEAP model-based analysis to low-carbon transformation path in the power sector: a case study of Guangdong–Hong Kong–Macao Greater Bay Area

**DOI:** 10.1038/s41598-024-57703-w

**Published:** 2024-03-28

**Authors:** Mengke Xu, Cuiping Liao, Ying Huang, Xiaoquan Gao, Genglin Dong, Zhen Liu

**Affiliations:** 1 School of Energy Science and Engineering, University of Science and Technology of China, Hefei, 230026 Anhui China; 2 Guangzhou Institute of Energy Conversion, Chinese Academy of Sciences, Guangzhou, 510640 Guangdong China; 3 Guangdong Provincial Key Laboratory of New and Renewable Energy Research and Development, Guangzhou, 510640 Guangdong China; 4 CAS Key Laboratory of Renewable Energy, Guangzhou, 510640 Guangdong China; 5 School of Engineering Science, University of Science and Technology of China, Hefei, 230026 Anhui China

**Keywords:** Carbon emission, LEAP, Power, Cost, Low-carbon transition, Sustainability, Energy management, Energy policy, Energy supply and demand

## Abstract

As a major carbon emitter, the power sector plays a crucial role in realizing the goal of carbon peaking and carbon neutrality. This study constructed a low-carbon power system based on the LEAP model (LEAP-GBA) with 2020 as a statistic base aiming of exploring the low-carbon transformation pathway of the power sector in the Guangdong–Hong Kong, and Macao Greater Bay Area (GBA). Five scenarios are set up to simulate the demand, power generation structure, carbon emissions, and power generation costs in the power sector under different scenarios. The results indicate that total electricity demand will peak after 2050, with 80% of it coming from industry, buildings and residential use. To achieve net-zero emissions from the power sector in the GBA, a future power generation mix dominated by nuclear and renewable energy generation and supplemented by fossil energy generation equipped with CCUS technologies. BECCS technology and nuclear power are the key to realize zero carbon emissions from the power sector in the GBA, so it should be the first to promote BECCS technology testing and commercial application, improve the deployment of nuclear power sites, and push forward the construction of nuclear power and technology improvement in the next 40 years.

## Introduction

With President Xi’s goal of “carbon neutrality”, achieving the dual-carbon target has become a major opportunity and challenge for China^1^. As one of the most open and dynamic regions in China, the GBA is an important spatial carrier for China to build world-class city clusters and participate in global competition^2^. Its green and low-carbon transformation and development has attracted much attention. Electricity, as an indispensable material basis for the production and life of modern society, is transformed from fossil energy, nuclear energy, renewable energy and other energy resources, and is the main way of non-fossil energy utilization. With socio-economic development and increasing electrification level, electricity consumption has been increasing year by year. Electricity consumption in the GBA in 2020 will reach 550.8 billion kWh, an increase of 56% compared with 2010, and the installed capacity of power generation will still be dominated by thermal power, which accounts for more than 75% of the total. The development of renewable energy within the GBA has been hindered, with the development of photovoltaics severely hampered by imperfect distributed photovoltaic mechanisms and unequal approval processes. Onshore wind power development is restricted by mountain protection policies^3^, and offshore wind power resources are limited. Hydropower is difficult to continue in-depth development due to resource endowment. The urbanization rate of the GBA in 2020 will have reached more than 85%, with fewer agricultural and forestry resources and limited land available, making it unsuitable for the development of biomass power plants based on agricultural and forestry wastes, and only waste incineration can be developed. 2020 average power generation standard coal consumption of thermal power units in the Greater Bay Area will be around 295 g of standard coal/kWh, which is a large gap compared with the national average of 287 g of standard coal/kWh^4^. Carbon emissions from the power sector in the GBA will account for 49.8% of the total carbon emissions from energy consumption in 2020, and carbon emission reduction in the power sector needs to be addressed urgently.

There have been many studies on low-carbon power transformation. The key to low-carbon power transition lies in the construction of a new type of power system with new energy as the main body, constituting a comprehensive energy service system centered on electricity. It is required to build new power systems that technological innovations, including efficient and low-cost solar and wind power generation technologies^5^, ultra-high voltage transmission and distribution technologies^6^, small reactor nuclear power plant technologies^7^, carbon capture technologies, energy storage technologies^8^ and smart grids^9^. However, the relationship between the scale of expansion of the new energy industry and technology should be balanced, taking into account the two dimensions of scale and technology in the process of energy transition^10^.

Considering the long construction period of power generation facilities, they need to be planned in advance. In recent years, research on power system planning simulation has been deepening. The studies mostly use integrated assessment models to analysis the decarbonization pathway of the power sector. They mainly include top–down macro prediction models and bottom-up micro prediction models. Typical macro forecasting model is CGE model. Xu Hongwei^11^, Wang Peng et al.^12^ simulated electricity demand and investment return under different paths in the process of low-carbon transition of electricity in GBA by CGE model. The approach is based on general equilibrium theory and is weak on analyses related to abatement technologies. Micro-prediction models include AIM Enduse, LEAP model, etc. Luo Yuejun et al.^13^ analyzed the impact of carbon emissions trading mechanism on electricity transition path based on AIM Enduse model. The model focuses on the analysis of the impact of a single technology or policy on overall carbon emissions. Compared with other models, the LEAP model can comprehensively evaluate the impacts of various technologies and policy measures on energy conservation and emission reduction in terms of the supply of energy structure, the level of energy technology, and the demand for energy, which is more suitable for analyzing the medium- and long-term scenarios of the power sector in this study. Nayyar^14^, Cai Liya^15^, and Nojedehi et al.^16^ forecasted the medium- and long-term demand for electricity using the LEAP model. Overall, most of the current domestic studies on low-carbon transition paths in the power sector are based on national, provincial and municipal studies, and there are fewer studies on the low-carbon transition of power in urban agglomerations. China’s regions have large differences in energy resource potential, and there is obvious regional heterogeneity in the structure of power generation and energy consumption, and the path of low-carbon transformation of electric power should be adapted to local conditions.

The innovation of this study is to consider the technology learning rate in the model calculation, the carbon capture rate of CCUS technology and the negative carbon emission factor of BECCS technology in the simulation process, and the power self-sufficiency rate and the energy self-sufficiency rate of power generation in the result analysis process. The significance of this paper is that the importance of nuclear power is demonstrated by the substitutability of nuclear power and renewable energy, the contribution of CCUS technology to the low-carbon transition is quantified by the carbon capture rate, and the security of local power generation is considered by the power self-sufficiency rate and the energy self-sufficiency rate of power generation. The results of the study can provide a reference for the government to formulate a carbon–neutral action plan.

This research study chooses the GBA as the research object, and uses the LEAP model as the research tool to simulate the power generation structure and energy consumption structure of the power sector in GBA, and predicts the carbon emissions of the power sector. This study combines energy planning and local electricity supply and demand with conversion in each municipality, considers the constraints of the local economy, population, and urbanization rate, sets the business-as-usual (BAU) scenario (scenario for maintaining the current development trajectory) as the baseline, four low-carbon transition scenarios, analyzes the electricity demand, generation structure, CO_2_ emissions, and generation costs under different scenarios, and explores the path to carbon neutrality in the power sector. This paper is structured as follows: “Introduction” introduces the research background and research methodology of the power sector low carbon transition study. “Scenario design” presents the research methodology and key assumptions used in this study. “Results and analysis” presents the methodology for setting up the various scenarios. “Discussion” provides a detailed analysis and discussion of the simulation results.

## Scenario design

According to *Guangdong Province’s 14th Five-Year Plan for Energy Development*^17^ and *Outline Development Plan for the Guangdong–Hong Kong–Macao Greater Bay Area*^18^, the future development of the GBA should optimize the energy structure, promote green and low-carbon energy transformation, and build a new type of power system, while improving the level of terminal electrification. This study sets up four low-carbon transition scenarios, clean energy generation (CEG) scenario, carbon capture, utilization and storage (CCUS) scenario, natural gas generation (NGE) scenario, and natural gas generation + carbon capture, utilization, and storage (NGE+CCUS) scenario, using the business-as-usual (BAU) scenario as a reference to analyze the carbon emissions, power generation costs, and power supply reliability of the power sector, and explore the best carbon neutral path in power sector for the GBA.

Electricity demand in the GBA is forecast to be 735 billion kWh in 2025, with an average annual growth rate of about 6% during the 14th Five-Year Plan period, which is basically in line with the 14th Five-Year Plan energy plans of various municipalities. With reference to the planning of key projects in Guangdong, Hong Kong and Macao Greater Bay Area municipalities in 2021, 2022 and 2023, and taking into account the longer construction cycle of generating units and the smaller scale of actual commissioning, the installed local power generation capacity in 2025 is lower than that of the 14th Five-Year Plan in this paper at the time of the scenario parameter setting.

### Business-as-usual scenario (BAU)

**Figure 1:**
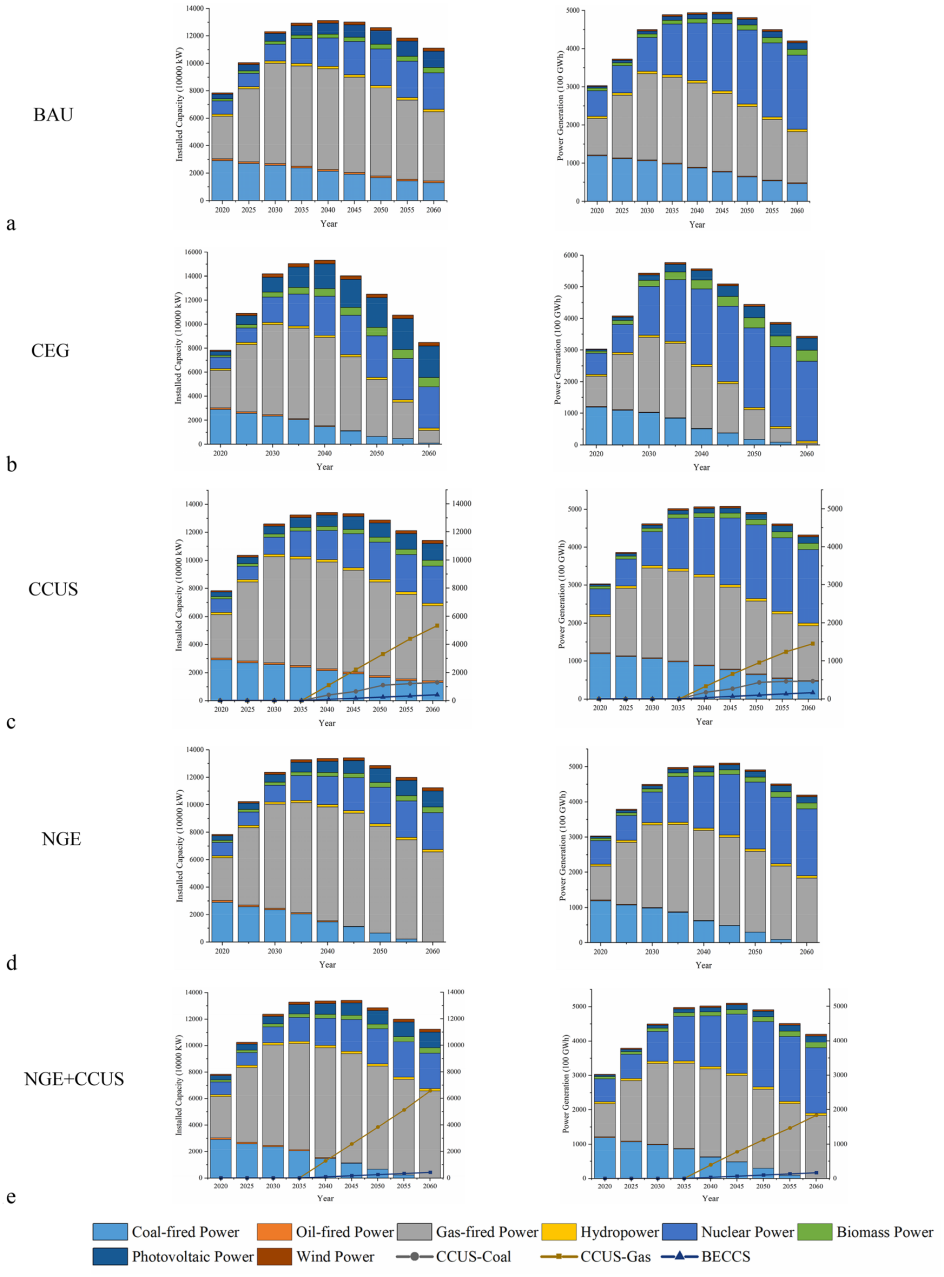
Installed capacity and generation structure in GBA under each scenario.

In the BAU scenario, the power sector’s installed capacity structure and generation structure are based primarily on the energy planning policies of the GBA administrative regions^19,20^ and no further carbon reduction measures are considered. In principle, there will be no new coal power generation and the old coal power generation units will be retired according to their service life, while gas power generation, renewable energy generation and nuclear power generation will be steadily developed. The key power projects under the 14th Five-Year Plan of each city will be completed, and some of the gas power generation units will be retired gradually after 2045 according to their service life. According to the relevant national standards, thermal power units within the GBA should complete energy-saving retrofits within the retrofit cycle^21–23^. Biomass power generation will be developed according to the existing planning and speed, with a waste incineration treatment capacity of about 159 kilo-tons per day, accounting for about 50% of the amount of waste to be treated. Photovoltaic power generation will be developed according to the current planning and growth rate, with an installed capacity of about 12 million kW to be completed by 2060, and wind and hydropower will be developed according to the existing planning and not further developed due to limited resources. Electricity demand is a continuation of existing policies and trends. The local installed generation capacity and generation structure under the BAU scenario are shown in Fig. 1a. By 2060, coal-fired, gas-fired, nuclear and renewable energy sources will account for 11.4%, 46.7%, 23.3% and 17.5% of the GBA’s local electricity generation capacity respectively.

### Low carbon transition scenario

This study establishes a low-carbon transition scenario based on the BAU scenario. In this scenario, other carbon reduction measures in addition to existing plans are considered. This study sets up four low-carbon transition scenarios according to different carbon reduction measures. It also compares the power generation costs and reliability of four low-carbon transition paths to identify the optimal low-carbon transition path.

#### CEG scenario

The scenario is set up to consider more clean energy generation and reduce carbon emissions from the power generation sector. Considering that CO_2_ emissions from the power sector are mainly from fossil fuel generation, the CEG scenario further promotes renewable energy generation and nuclear power and retires coal-, gas- and oil-fired power plants year by year according to their lifetimes. In this scenario, to achieve 100% clean energy generation and further promote nuclear power construction, the Daya Bay Nuclear Power Station in Shenzhen adopts new nuclear units to replace older units. The total installed capacity of nuclear power in the GBA will reach 34.5 million kW in 2060. Considering the economic development, the future direction of solar power generation in GBA is mainly in rooftop PV. According to each city planning and research, the installed PV power generation in the current plan is only 50% of the buildable installed capacity in GBA, so solar power generation is further promoted based on existing policies. The scenario sets up an annual growth of 56 MW in installed PV capacity, reaching 26.24 GW by 2060. Due to the resource endowment, both wind power and hydropower in GBA are developed according to the existing plan, and the installed wind power and hydropower in 2060 are 2.85 GW and 1.78 GW, respectively. According to the forecast of urban domestic waste generation per capita and population, the domestic waste volume in GBA in 2060 can reach 364 kilo-tons per day, setting the incineration treatment ratio at 80%^24^, and the installed power generation capacity can reach 7.75 GW. This scenario retains only a small amount of coal and gas power as backup units by 2060. By 2060, coal-fired power, gas-fired power, nuclear power, and renewable energy power will account for 0.2%, 1.5%, 73.5%, and 24.8% respectively in local power generation in GBA. The structure of installed capacity and generation under the CEG scenario are shown in Fig. 1b.

#### CCUS scenario

The commercial deployment of CCUS technology is critical to achieving carbon neutrality in the power sector. This scenario is based on the BAU scenario deploying CCUS technology on a unit-size basis after 2035, with all retained coal-fired and gas-fired power plants covered by 2060. BECCS technology has a carbon-negative role, and is deployed in this scenario after 2035, assuming that 150,000 kW of new BECCS installed capacity is added each year, covering all biomass generating units by 2060. The carbon emission factor for BECCS technology is set at − 3.214^25^. The carbon capture rate for CCUS-coal and CCUS-gas generation is 85%^26^. By 2060, CCUS-coal-fired, CCUS-gas-fired, nuclear and renewables will account for 10.8%, 33.5%, 44.9% and 10.3% of GBA’s local electricity generation, respectively. The installed local generation mix and the local generation mix in the CCUS scenario are shown in Fig. 1c.

#### NGE scenario

Natural gas is generally regarded as a clean fossil energy source that emits less carbon than other fossil fuels and reduces the production of other air pollutants significantly. In this scenario based on the BAU scenario, natural gas-fired power generation is the main form of power generation in GBA, the merit order is adjusted from 2 to 1, the coal and oil power units are gradually retired, and the incentive for gas-fired power generation is stimulated through policies such as improving the natural gas price mechanism. By 2060, local power generation in this scenario mainly consists of natural gas-fired power generation, renewable power generation, and nuclear power generation, with the proportion of 43.7%, 45.2%, and 11.1% respectively. The structure of installed local generation and local generation capacity in the NGE scenario are shown in Fig. 1d.

#### NGE + CCUS scenario

The natural gas power generation process still generates some carbon emissions. Therefore, the NGE+CCUS scenario is set based on the NGE scenario. The CCUS technology is deployed gradually after 2035 according to the size of natural gas power plants, with an average of 2.5 million kW of new CCUS-natural gas-fired generation capacity per year, covering all natural gas-fired power plants by 2060. The structure of installed local generation and local generation capacity under the NGE+CCUS scenario is shown in Fig. 1e.

## Results and analysis

This study predicts the future electricity demand and carbon emissions in GBA through the LEAP model and designs four low-carbon transition scenarios. The four power supply scenarios are compared in terms of carbon emission, power supply reliability, and cost to find out the optimal low-carbon transition path for the power sector in GBA.

### Electricity demand forecast

**Figure 2:**
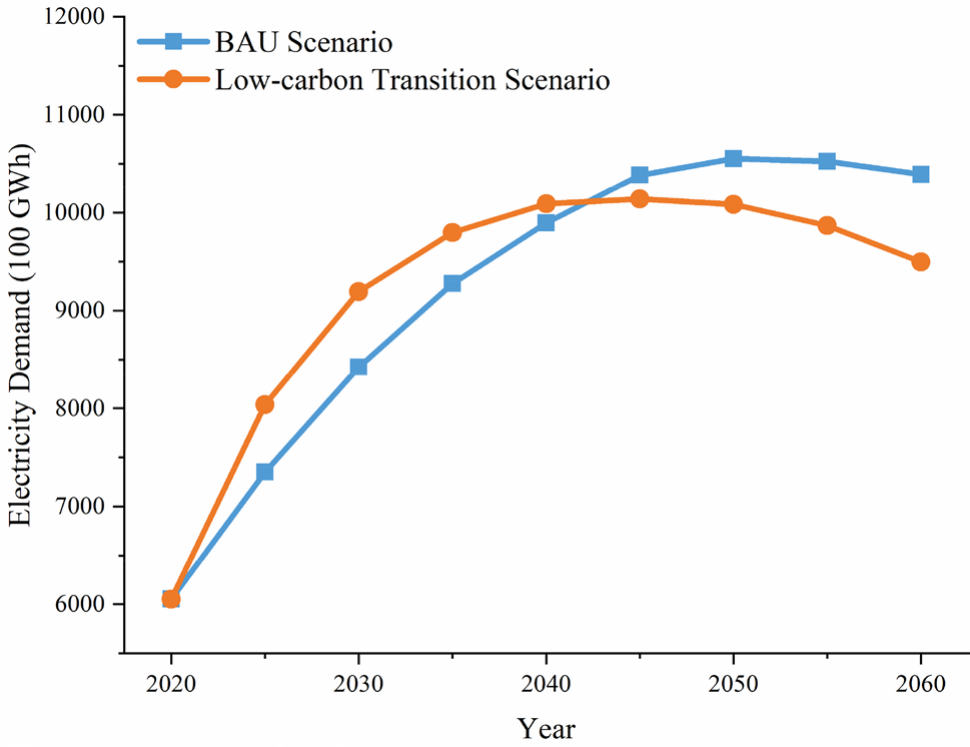
Total electricity demand forecasts.

**Figure 3:**
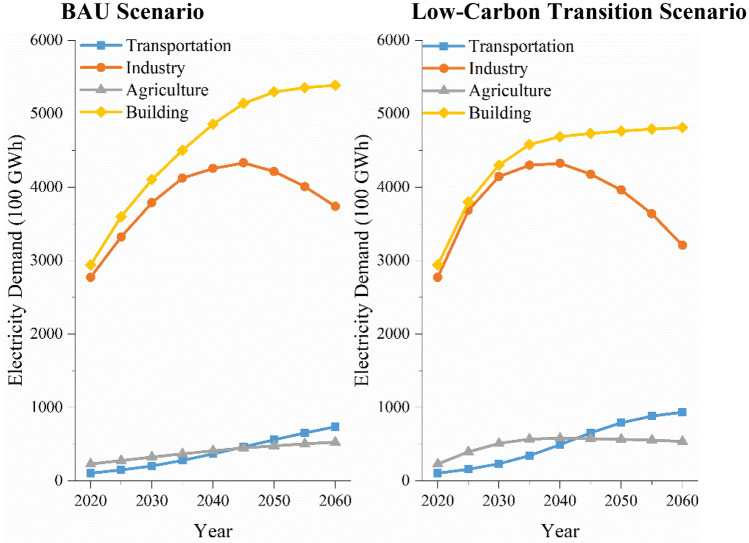
Electricity demand forecasts by scenario sub-sector.

The forecast results of total electricity demand and that by sector in GBA from 2020 to 2060 are shown in Figs. 2 and 3. It is in a period of rapid development for GBA from 2020 to 2060. With the rapid growth in population, GDP, and electrification levels and the level of energy efficiency of equipment across all sectors, electricity demand in the GBA peaks at 1055.8 billion kWh in 2050 under the BAU scenario, and it peaks at 1029.8 billion kWh in 2040 under the Low-carbon transition scenario. Comparing the electricity demand in different scenarios, the BAU scenario peaks in 2055. The low-carbon transition scenario has its peak shifted forward to 2050, and its electricity demand maximum is lower than that in the BAU scenario. This is largely attributed to increased energy efficiency levels in consumer-side devices. As can be seen from Fig. 3, the electricity demand mainly comes from the industrial sector and the construction sector. Electricity demand in the transportation sector increases year by year in both the BAU scenario and the low carbon transition scenario. Unlike the BAU scenario, its growth rate slows down after 2035 in the low-carbon transition scenario. This is due to the reduction in unit electricity consumption resulting from further improvements in electric vehicle technology and energy efficiency in the low-carbon transition scenario. In the BAU scenario, industrial sector electricity demand peaks at 433.2 billion kWh in 2045, and it peaks at 432.6 billion kWh 5 years ahead of schedule due to the accelerated pace of implementation of energy efficiency policies and the accelerated upgrading of energy efficiency levels in the low-carbon transition scenario. Electricity demand in the building sector increases annually in both scenarios, but it slows down significantly after 2035 in the low-carbon transition scenario. In the BAU scenario, agricultural electricity demand has been on an upward trend. The low-carbon transition scenario, on the other hand, accelerates energy efficiency and promotes high-tech and green agriculture, whose electricity demand peaks in 2035 and then begins to decline annually.

### Sensitivity analysis

#### Sensitivity analysis of electricity supply and demand

**Figure 4:**
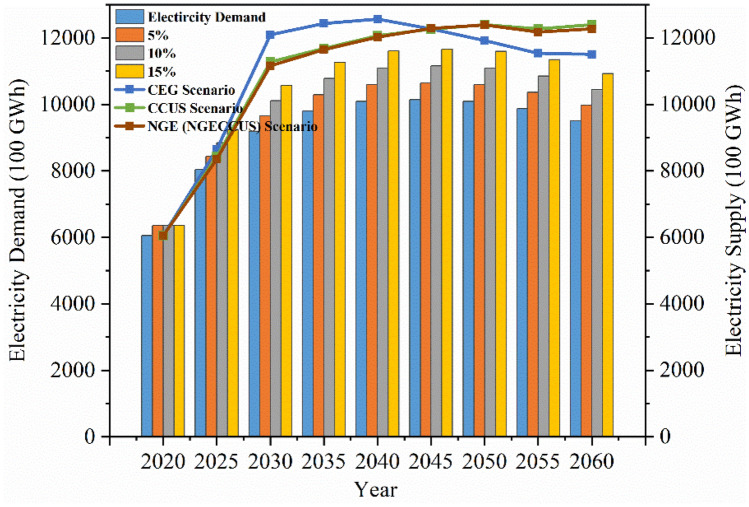
Balance analysis of electricity supply and demand in GBA.

Electricity demand fluctuations will challenge the security of GBA’s electricity supply, due to the impact of uncertainty factors, there are fluctuations in electricity demand, and sensitivity analysis should be carried out on the balance of electricity supply and demand. In 2020, the GBA purchased power outside the province of 320 billion kWh, 140 billion kWh of which came from hydropower, assuming that the total amount of electricity purchased outside the province will remain unchanged in the future. Purchase of electricity from outside the province mainly from the neigh-boring cities in the east, north and west of Guangdong, Yangjiang six sets of 1.25 million kW of nuclear power in the end of 2019, all completed and put into operation, Shanwei Lufeng nuclear power plant six sets of 1.25 million kW of nuclear power units, Haifeng nuclear power plant eight sets of 1 million kW of nuclear power units, Shaoguan nuclear power plant four sets of 1.25 million kW of nuclear power units are in the planning of the construction. It is expected that by 2060, 26.98 million kW of nuclear power units will be put into operation to ensure the supply of electricity, generating 200 billion kWh of electricity annually. According to the *Offshore Wind Power Development Plan of Guangdong Province *(*2017–2030*), in the eastern and western regions of Guangdong, the installed capacity in the offshore shallow water area is 8.35 million kW, and the installed capacity in the offshore deep water area is 57 million kW, with a total installed capacity of up to 65 million kW, and a total power generation of up to 200 billion kWh. In the coastal areas of eastern and western Guangdong, a series of large-scale coal-fired units with a total installed capacity of about 18 million kW have been built because of their geographical advantages. There are also some large coal-fired units under construction in key planning projects in Guangdong Province. It is expected that by 2025, the installed capacity of large coal-fired units in eastern and western Guangdong will reach 20 million kW, providing 80 billion kWh of electricity. Assuming that end-use electricity consumption is raised by 5%, 10%, and 15% respectively under the low-carbon scenario, an analysis of the balance of electricity supply and demand in the GBA is shown in Fig. 4.

Within the 5% confidence interval, only the CEG and CCUS scenarios basically meet electricity demand, and within the 10% and 15% confidence intervals, an additional 50 billion kWh and 90 billion kWh of out-of-province power purchases are required in 2025 to meet electricity demand under the low-carbon scenario.

#### Sensitivity analysis of installed nuclear power capacity to electricity generation

**Figure 5:**
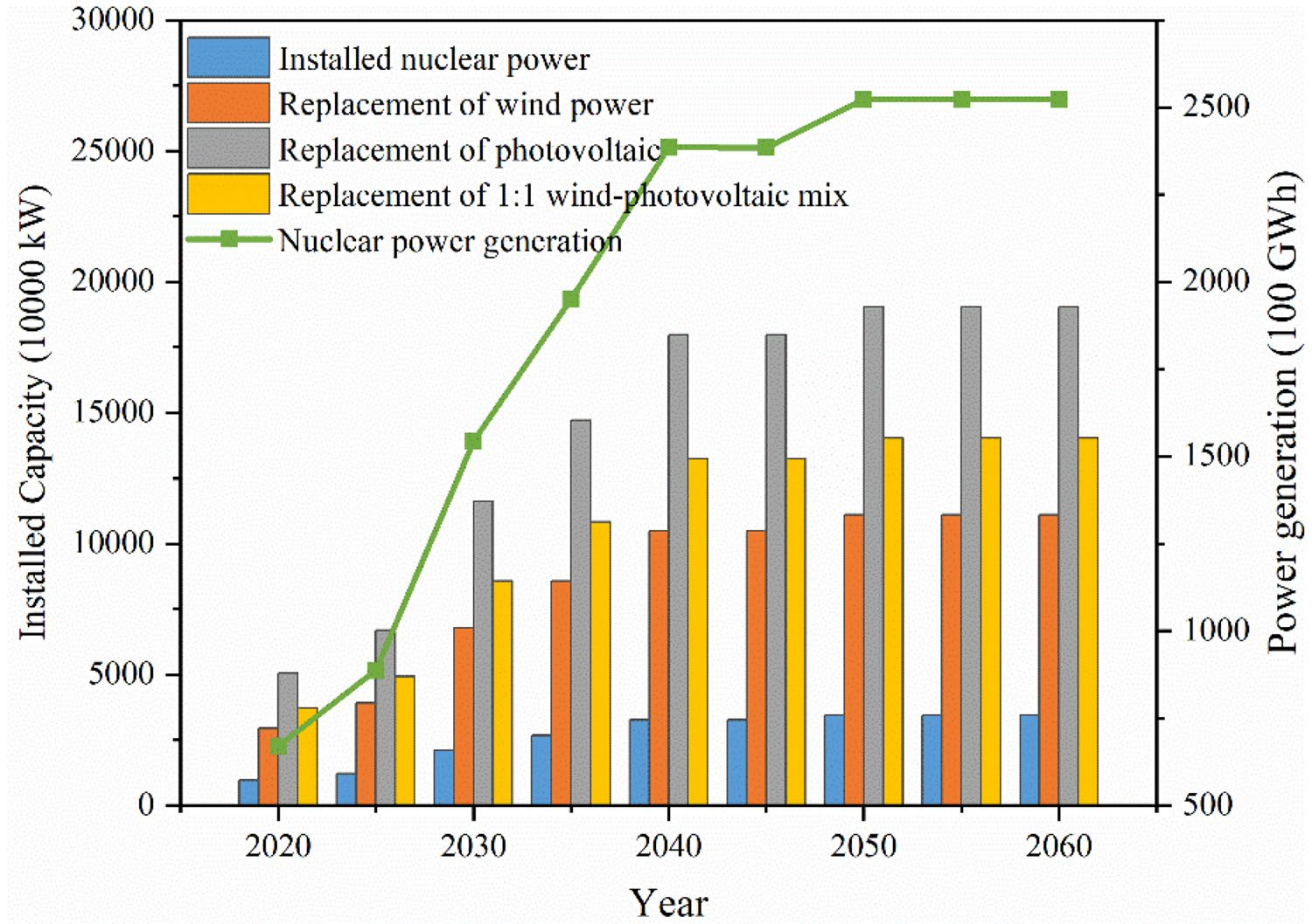
Electricity equivalent nuclear and renewable energy installed substitution.

**Figure 6:**
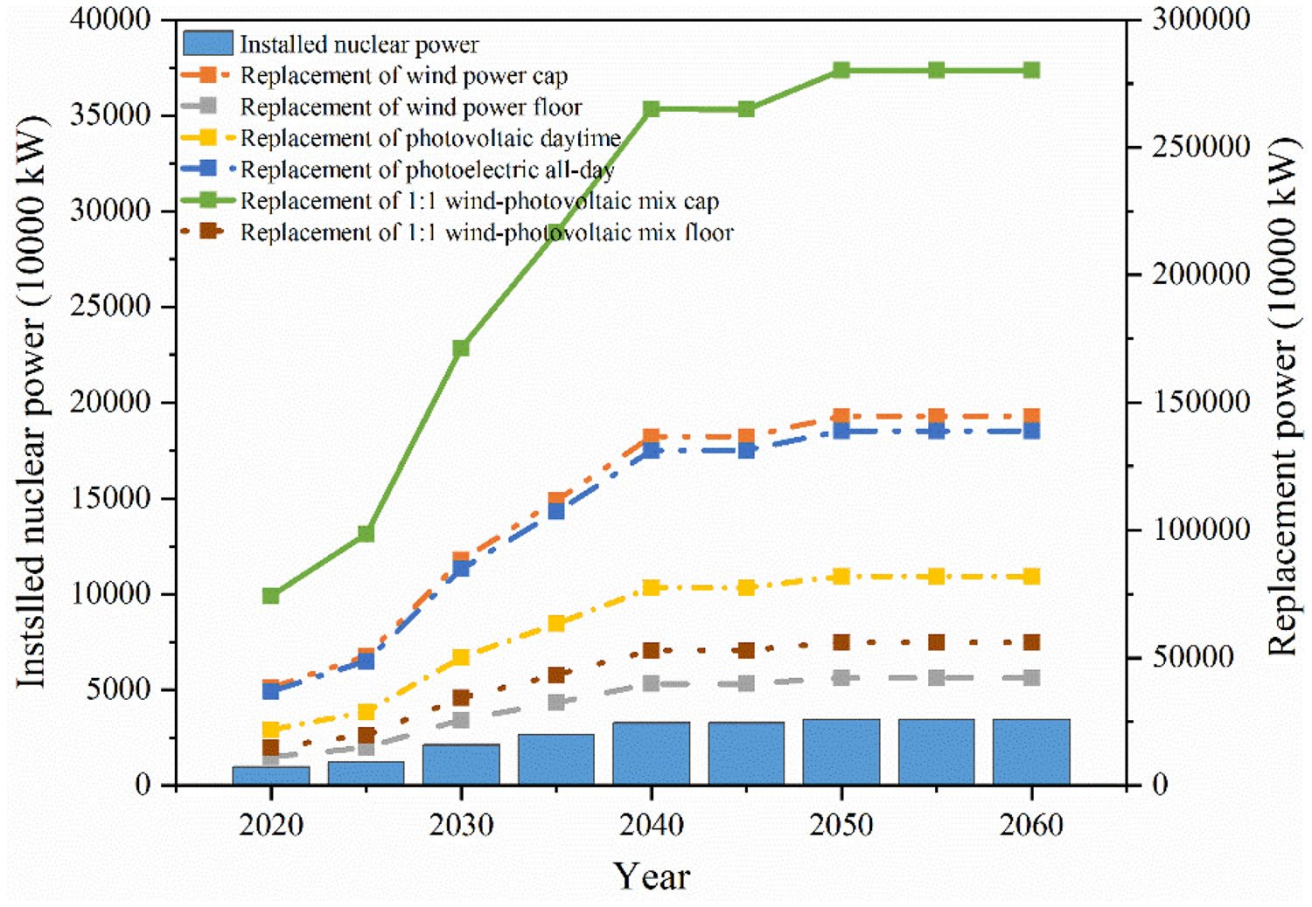
Capacity-equivalent nuclear and renewable energy installations.

Nuclear power, as clean electricity, is able to maintain a stable high output and is an important way to replace fossil energy sources. This paper analyses the installed amount of renewable energy that can be replaced by nuclear power installation according to the principle of power equivalence as shown in Fig. 5. It is expected that 34.5 million kW of nuclear power will be commissioned in the GBA by 2060, which can replace 190.2 million kW of photovoltaic power generation or 111.0 million kW of wind power, discounted on the basis of the 2020 average annual power generation hours of wind power and solar power. Considering the complementary nature of wind–photovoltaic power generation, 140.2 million kW can be replaced by a 1:1 wind-photovoltaic mix. The amount of electricity reflects the effective generation time, the capacity reflects the generation capacity, and the reliability of renewable energy generation is low. The upper and lower confidence limits of wind power capacity are 26.37% and 7.68% respectively^27^, and the confidence limits of photovoltaic capacity are 13.7% and 23.2% considering the daytime and all-day period respectively^28^, and the upper and lower confidence limits of wind and photovoltaic integrated capacity are 25% and 5% respectively^29^. The new energy installed capacity that can be replaced by nuclear power with capacity equivalence is shown in Fig. 6. According to the principle of capacity substitution, in 2060, the installed nuclear power capacity in the GBA can replace the upper limit of 2.8 billion kW and the lower limit of 560 million kW of wind-photovoltaic mix power generation, which is a considerable benefit.

### Carbon emission prediction

**Figure 7:**
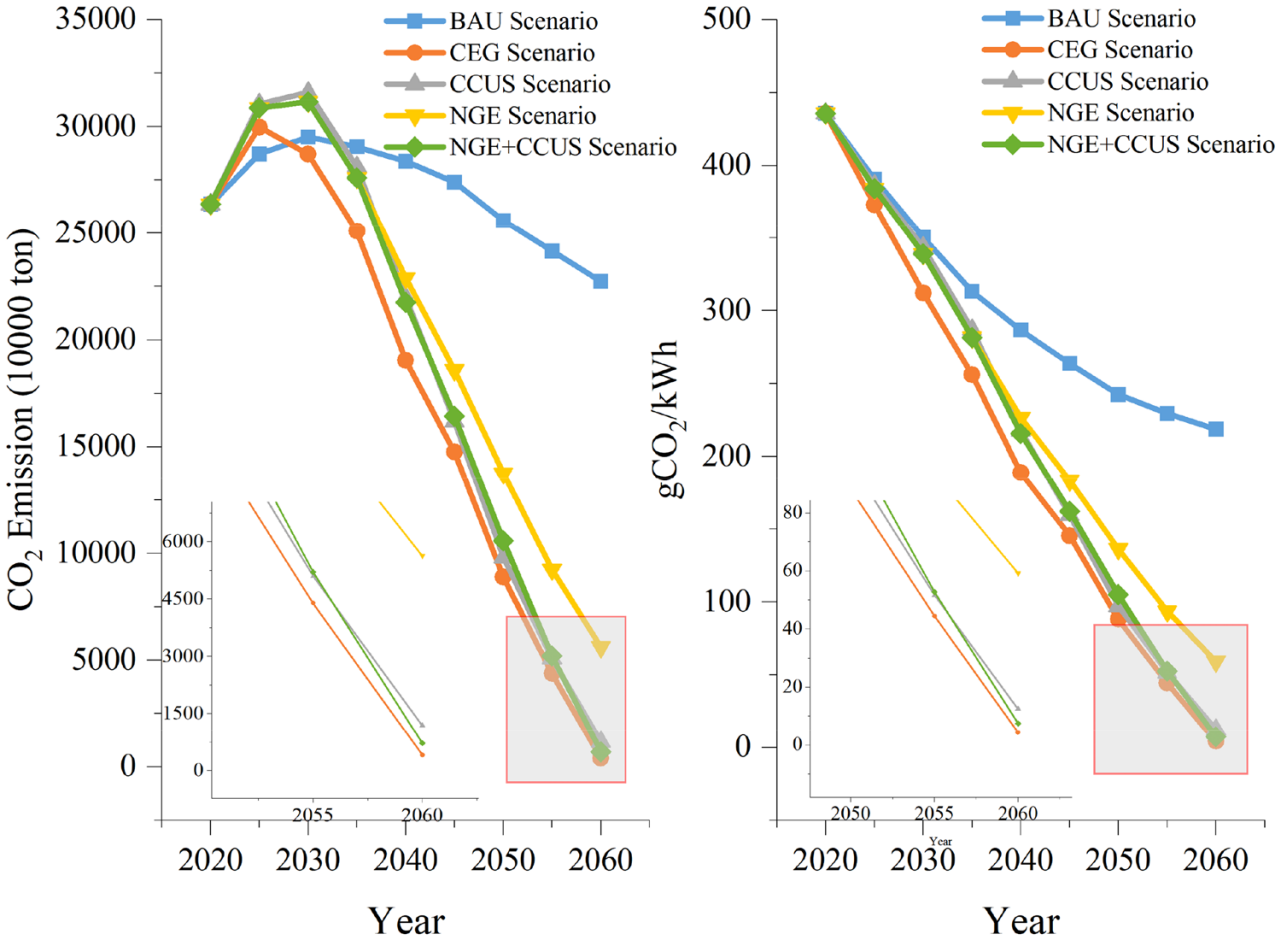
CO_2_ emission prediction for each scenario.

In this study, the energy consumption of power generation is calculated based on the power generation structure of the power sector, and the carbon emissions of the power sector in GBA are calculated based on that. The carbon emission factor of purchased electricity adopts the average carbon emission factor of the power grid in Guangdong Province. The carbon emission projections under each scenario are shown in Fig. 7. Carbon emissions in the BAU scenario decline slowly each year after peaking at 295.13 million tons in 2030, to 227.32 million tons in 2060. The CO_2_ emissions per unit of electricity generation will decline annually to 218.75 g/kWh by 2060. Therefore, it is difficult to achieve net-zero emissions in the power sector under the current policy trend. Carbon emissions in the CEG scenario peak at 299.76 million tons in 2025 and then decline each year to 4.05 million tons in 2060. And the CO_2_ emissions per unit of electricity generation declining annually to 4.26 g/kWh by 2060. Therefore, enhanced implementation of clean energy policies could lead to an earlier peak in carbon emissions and a 98% reduction in power sector carbon emissions by 2060. Carbon emissions in the CCUS scenario peak at 315.99 million tons in 2030 and then decline each year to 11.80 million tons in 2060. And the CO_2_ emissions per unit of electricity generation declining annually to 12.42 g/kWh by 2060. So consideration of CCUS deployment could reduce carbon emissions in 2060 but also increase peak carbon emissions. Carbon emissions in the NGE scenario peak at 311.68 million tons in 2030 and then decline each year to 56.25 million tons in 2060. The CO_2_ emissions per unit of electricity generation declining annually to 59.22 g/kWh by 2060. Carbon emissions in the NGE+CCUS scenario peak at 311.68 million tons in 2030 and then decline each year to 7.11 million tons in 2060. And the CO_2_ emissions per unit of electricity generation declining annually to 7.49 g/kWh by 2060. So considering the coal-to-gas policy reduces carbon emissions less, while considering that CCUS deployment can reduce carbon emissions by 96.9% in 2060, which is lower than the carbon reductions in the CEG scenario. The percentage of CO_2_ emission reduction under each scenario is shown in Table 1. The simulation results show that accelerating the decommissioning of coal-fired power plants while accelerating the application of CCUS technology and promoting renewable power generation and nuclear power generation can help achieve carbon neutrality goals for the power sector in GBA.

**Table 1:** The percentage of CO_2_ emission reduction under each scenario.

	Year	BAU	CEG	CCUS	NGE	NGE+CCUS
Parameters CO_2_ emission reduction percent (%)	2020	–	0.00	0.00	0.00	0.00
	2025	–	− 4.38	− 8.16	− 7.47	− 7.47
	2030	–	2.74	− 7.07	− 5.61	− 5.61
	2035	–	13.63	3.23	5.00	5.00
	2040	–	32.89	22.57	19.35	23.33
	2045		46.14	40.92	32.12	39.99
	2050		65.26	61.68	46.28	58.63
	2055		81.86	78.92	61.77	78.48
	2060		98.22	94.81	75.25	96.87

### Reliability analysis

This study also considers the reliability of electricity supply under different scenarios. The power sector supply reliability *δ* can be calculated from the electricity self-sufficiency rate *λ* and the energy self-sufficiency rate *μ* of power generation. It can be expressed as follows:

1 $$\delta = \lambda \cdot \mu$$

which are shown in Table 2.

**Table 2:** Sustainability and security parameters under each scenario.

Parameters	Year	BAU	CEG	CCUS	NGE	NGE+CCUS
Electricity self-sufficiency level (*λ*) (%)	2020	50.06	50.06	50.06	50.06	50.06
	2030	54.79	59.09	50.17	48.91	48.91
	2040	51.21	55.21	50.21	49.77	49.77
	2050	46.62	44.04	48.78	48.69	48.69
	2060	41.59	36.15	45.50	44.21	44.21
Energy self-sufficiency level of power generation (*μ*) (%)	2020	55.44	55.44	55.44	55.44	55.44
	2030	57.9	68.79	57.9	54.29	54.29
	2040	64.05	80.46	63.79	58.04	55.81
	2050	67.61	90.94	60.37	59.14	52.80
	2060	67.96	100	56.63	56.68	47.23
Electricity reliability (*δ*)	2020	27.76	27.76	27.76	27.76	27.76
	2030	31.72	40.65	29.05	26.55	26.55
	2040	32.80	44.42	32.03	28.89	27.78
	2050	31.52	40.05	29.45	28.80	25.71
	2060	28.26	36.15	25.77	25.06	20.88

In the BAU scenario, the *λ* in GBA decreases to 41.59% in 2060 while the *μ* increases to 67.96%. The *δ* increases from 27.76% in 2020 to 28.26% in 2060. In the CEG scenario, the *λ* in GBA decreases to 36.15% in 2060, which is due to the limited availability of renewable energy resources and the difficulty of nuclear power siting, while the *μ* increases to 100%. So the *δ* increases from 27.76% in 2020 to 36.15% in 2060. In the CCUS scenario, the *λ* in GBA decreases to 45.50% in 2060, which is due to the impact of energy prices, and the *μ* increases to 56.63%. So the *δ* decreases from 27.76% in 2020 to 25.77% in 2060. In the NGE scenario, the *λ* in GBA decreases to 44.21% in 2060, which is due to the natural gas price and geographical resource constraints, and the *μ* increases to 56.68%. So the *δ* decreases from 27.76% in 2020 to 25.06% in 2060. In the NGE+CCUS scenario, the *λ* in GBA decreases to 44.21% in 2060 and the *μ* decreases to 47.23%. The *δ* decreases from 27.76% in 2020 to 20.88% in 2060. This indicates that both the deployment of CCUS technology and the conversion of coal power to gas power will reduce the reliability of the electricity supply and that policies to promote clean energy generation should be accelerated.

### Cost of electricity generation analysis

**Figure 8:**
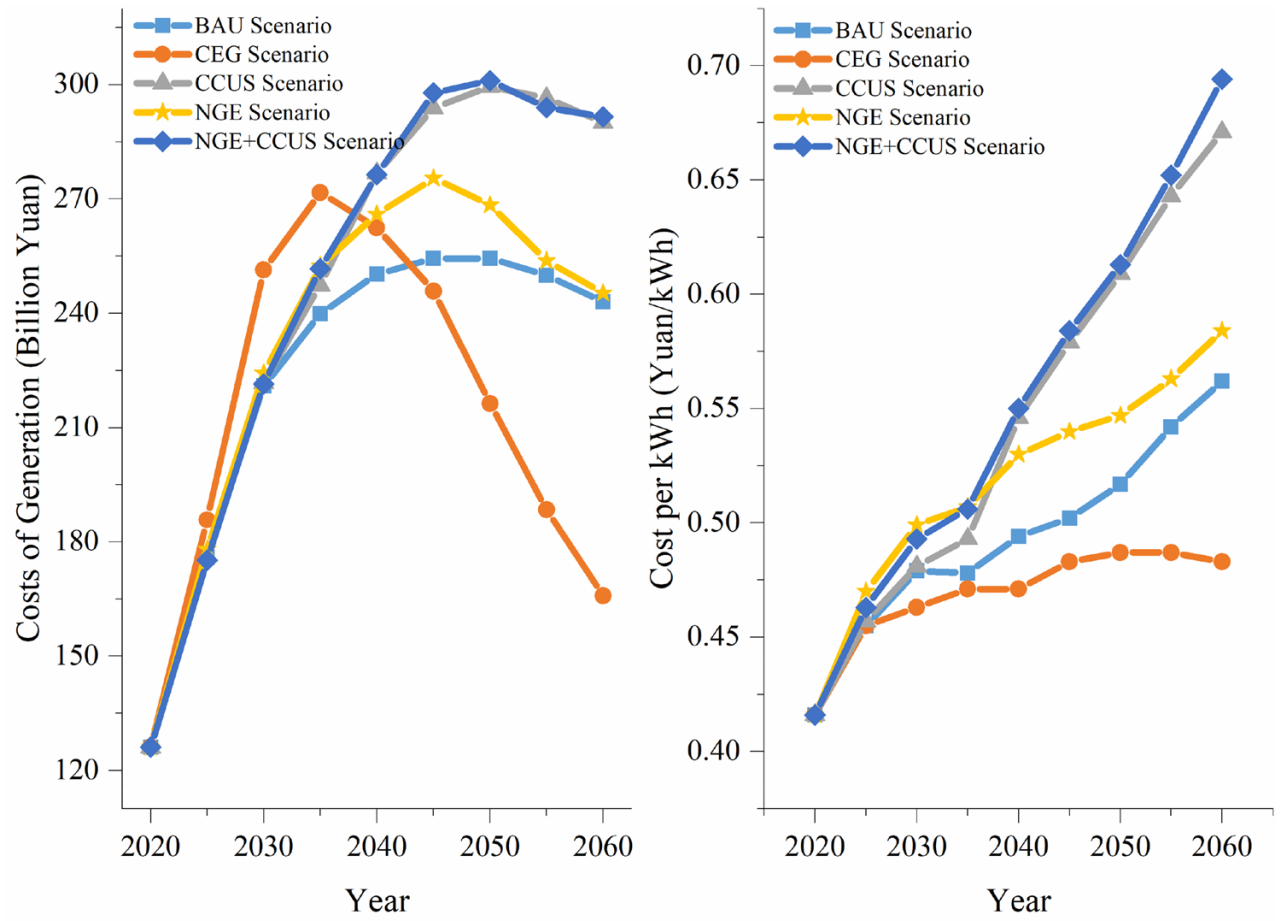
Power generation cost under different scenarios.

In this study, the total power generation cost and the unit power generation cost under different scenarios are shown in Fig. 8 and Table 3. In the BAU scenario, the total cost of generating electricity peaks at 254.40 billion yuan in 2050 and then declines each year to 243.02 billion yuan in 2060. And the cost per unit of electricity generation increased yearly to 0.56 yuan/kWh. In the CEG scenario, the total cost of generating electricity peaks at 271.70 billion yuan in 2035 and then declines each year to 165.87 billion yuan in 2060. The cost per unit of electricity generation peaks at 0.49 yuan/kWh in 2050 and then declines yearly to 0.48 yuan/kWh, which is 14.10% lower than that in the BAU scenario for 2060. In the CCUS scenario, the total cost of generating electricity peaks at 296.51 billion yuan in 2050 and then declines yearly to 290.10 billion yuan in 2060. The cost per unit of electricity generation increased yearly to 0.67 yuan/kWh, which is 19.37% higher than that in the BAU scenario for 2060. In the NGE scenario, the total cost of generating electricity peaks at 275.44 billion yuan in 2045 and then declines yearly to 245.31 billion yuan in 2060. The cost per unit of electricity generation increased yearly to 0.58 yuan/kWh, which is 3.89% higher than that in the BAU scenario for 2060. In the NGE+CCUS scenario, the total cost of generating electricity peaks at 301.04 billion yuan in 2050 and then declines yearly to 291.56 billion yuan in 2060. The cost per unit of electricity generation increased yearly to 0.69 yuan/kWh, which is 23.48% higher than that in the BAU scenario for 2060. The simulation results show that an increase in clean energy generation can be achieved by controlling fuel costs in a way that reduces generation costs, while the deployment of CCUS increases both fuel costs and the O&M costs of power equipment.

**Table 3:** Cost per kWh under different scenarios (yuan/kWh).

Year	BAU	CEG	CCUS	NGE	NGE+CCUS
2020	0.42	0.42	0.42	0.42	0.42
2025	0.46	0.46	0.46	0.47	0.46
2030	0.48	0.46	0.48	0.50	0.49
2035	0.48	0.47	0.49	0.51	0.51
2040	0.49	0.47	0.55	0.53	0.55
2045	0.50	0.48	0.58	0.54	0.58
2050	0.52	0.49	0.61	0.55	0.61
2055	0.54	0.49	0.64	0.56	0.65
2060	0.56	0.48	0.67	0.58	0.69

## Discussion

Electricity demand mainly comes from industry, buildings, and residential life, accounting for more than 80% of the total electricity demand. So increased electrification of the industry sector and the building sector is the key to reducing electricity demand in the GBA. Due to the instability of natural gas prices, gas-fired power is less economical and should not be the dominant power generation technology. Considering the local natural resource endowment of the GBA, wind- and hydroelectric power generation are also highly restricted. Therefore, accelerating the decommissioning of coal power plants and promoting the development of solar power, biomass power, and nuclear power are key to achieving carbon neutrality in the power sector of the GBA.

Large-scale renewable energy generation into the grid will bring greater volatility to the grid, the later O&M costs greatly increase, so the abandoned wind, abandoned light phenomenon is serious currently. Smart grid technology and energy storage technology can increase the flexible scheduling of electricity, maintain grid stability, and reduce the operation and maintenance costs of the grid. At present, both the State Grid and the Southern Power Grid have built a strong smart grid. Energy storage technology still has certain technical barriers and has a greater impact on the cost of power generation after large-scale application. Electricity-hydrogen integration is still in the technology demonstration stage. Research on the impact of smart grid technology and energy storage technology on power generation costs will be carried out in the follow-up research.

CCUS technology plays an important role in carbon emission reduction in the power sector. However, due to high cost and energy consumption, CCUS technology is still in the demonstration and promotion stage due to a lack of experience in large-scale demonstration projects. Moreover, the complete industrial chain of carbon dioxide capture, storage, and utilization has not yet been formed, which prevents the combination of carbon-emitting enterprises and carbon-demanding enterprises from forming an integrated CCUS model. The carbon cycle of the power sector of GBA should be realized in combination with the good storage conditions of the submarine saltwater layer in Guangdong Province and the good foundation of gas hydrate extraction technology of carbon dioxide replacement.

As a policy tool to push enterprises to make energy-saving and emission-reduction transitions, carbon market trading is playing an increasingly important role. The carbon market is efficient and flexible but requires a complex set of institutional designs to support it. The core of the carbon trading market is carbon pricing, and the impact of carbon pricing on carbon emission reduction and power generation costs in the power sector will also be continued in the follow-up research.

## Conclusions and recommendations

This study examines the ways to achieve carbon neutrality in the power sector in the GBA, a city cluster in the Pearl River Delta of China. This study adopts the LEAP model to construct a low-carbon power system in the GBA. The electricity demand in the GBA from 2020 to 2060 is analyzed from the demand side, and the power supply, power generation cost and CO_2_ emission of the power sector under different scenarios from 2020 to 2060 are analyzed from the supply side. The results of the scenario analyses are available for policy makers.

The total electricity demand in the GBA will increase annually over the next 20–30 years and then decrease annually as energy efficiency levels improve. Electricity demand in the industrial sector will peak in 2040–2045 and then gradually begin to decline, and building and residential electricity use will grow slowly through 2060 with no inflection point. As electricity demand grows, an additional 40 billionkWh of out-of-province electricity should be purchased in 2025 to meet electricity demand under the 10% confidence interval and to ensure security of supply.

Carbon emissions from the power sector vary under different scenarios. Under the BAU Scenario, CEG Scenario, CCUS Scenario, NGE Scenario, and NGE+CCUS Scenario, the CO_2_ emissions per unit of electricity generation are 219 g/kWh, 4 g/kWh, 12 g/kWh, 59 g/kWh, and 7 g/kWh by 2060, respectively. Accelerated decommissioning of coal plants, coal-to-gas conversion, and CCUS technology can all significantly reduce carbon emissions from the power generation sector. However, due to the limitation of carbon capture rate, CCUS technology cannot achieve zero carbon emission. Only through the development of nuclear power and renewable energy power generation can we truly realize zero carbon emissions from the power sector. Considering the volatility of power demand, a small amount of fossil power generation should be retained as a standby unit to ensure the reliability of power supply in the power sector. To ensure net-zero emissions from the power sector, all fossil energy generation and biomass generation should adopt CCUS and BECCS technologies by 2060.

Generation costs vary across scenarios. Deployment of CCUS technologies increases energy consumption costs and O&M costs in the power sector, and an increased share of natural gas generation raises fuel costs. Unit generation costs are highest in the NGE+CCUS scenario, reaching 0.69 yuan/kWh by 2060. The modeling results suggest that accelerating the retirement of thermal power plants and promoting clean energy generation can help reduce the cost of power generation. Meanwhile, the study considers the reliability of the power sector. Due to the limited use of renewable energy and the development of nuclear power in the GBA, a small amount of fossil power generation should be retained to improve the reliability of power supply, and CCUS technology should be adopted to reduce carbon emissions.

### Recommendations

#### Strengthening demand-side management of electricity

Between 2010 and 2020, terminal electricity consumption, per capita electricity demand and electricity consumption per unit of GDP in the GBA have increased year by year and electricity consumption per unit of GDP is higher than the level of international power-saving countries, so in the future, we should promote the decoupling of economic growth and electricity consumption by improving the ladder tariffs, valley-peak tariffs and smart grids etc., which can vigorously promote the demand-side management of electricity.

#### Strict control of coal power installation

The Central Government’s working opinion on the implementation of carbon neutrality suggests that fossil energy consumption should be strictly controlled, the pace of coal reduction should be accelerated, and the growth of coal consumption should be strictly controlled during the Fourteenth Five-Year Plan period and gradually reduced during the Fifteenth Five-Year Plan period. As the main source of carbon emissions in the power sector, coal power should be integrated into the future development process to maintain supply and peak adjustment, strictly control the addition of coal power units, and gradually retire old coal power units, accelerate the energy-saving upgrading and flexibility transformation of existing coal power units, and gradually transform coal power from the main force of power generation to a standby unit, and progressively reduce or even prohibit the bulk burning of coal.

#### Properly addressing the relationship between carbon neutrality in the long term and the development of natural gas in the near term

In recent years, the GBA has been actively encouraging the application of natural gas, actively developing natural gas-fired power generation, identifying natural gas sources and gradually constructing and improving the natural gas pipeline network, and upgrading the reception, transmission and distribution capacity of the trunk pipeline network. Natural gas-fired power generation, with its flexible operation, short start-up and stopping time and fast climbing rate, is a necessary support for the large-scale development of renewable energy. Therefore, the relationship between the long-term carbon neutral target and the near-term transition of natural gas power generation should be properly handled, the natural gas price mechanism should be improved, and appropriate laws, regulations and policy documents should be introduced when necessary to stimulate the power generation enthusiasm of natural gas units.

#### Promoting cleaner purchased electricity

In the next 40 years, it should continue to broaden the green channel for clean power from outside the region to enter the Bay, sign long-term strategic agreements, build a cross-provincial and cross-regional power transmission system for foreign clean power, and enhance the ability of clean energy bases to transmit power. Accelerate the promotion of inter-regional investment in renewable energy power plants by municipalities in the GBA, and tap the clean energy resources of the province's eastern, northern and western Guangdong.

#### Promoting energy efficiency of thermal power generating units in the GBA

The thermal power units in the GBA started early and are old, and the standard coal consumption per unit of power generation is higher than the domestic average. It is possible to optimize the energy system and improve the energy efficiency level of the units by designating measures for a special action program for energy efficiency improvement, increasing green financial expenditures^30^, upgrading the management level of energy efficiency indicators, and improving the unit's online performance calculation, indicator and consumption analysis system.

## Methodology

The Low Emissions Analysis Platform (LEAP) model, developed by the Stockholm Environment Institute, is widely used for energy policy analysis and climate change mitigation assessment. It is based on scenario analysis with built-in energy, emissions, and cost–benefit accounting, allowing a comparison of energy demand, social costs and benefits, and environmental impacts under different scenarios.

### LEAP framework and basic data

**Figure 9:**
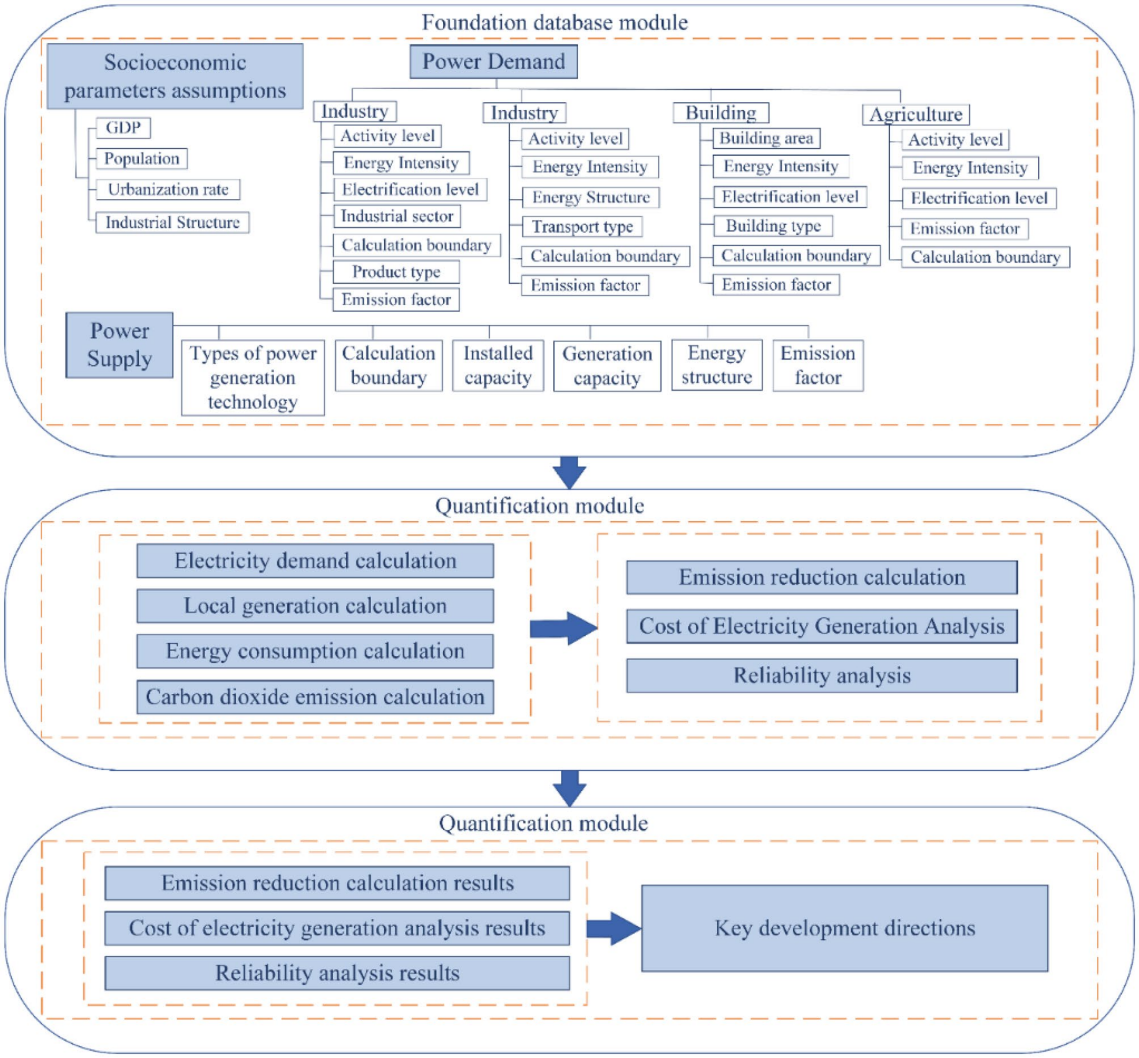
Power planning model structure of GBA.

This study analyzes the pathway to carbon neutrality in the power sector of the GBA by 2060. The study consists of five modules: key assumptions, power demand, power supply, CO_2_ emission, and power generation cost. The key assumptions module mainly includes GDP, population, urbanization rate, and industrial structure. The power demand module is used to simulate the electricity consumption structure on the demand side of GBA, which includes four sectors: industry, transportation, construction, and agriculture. The power supply module simulates the power installation structure and power generation structure on the supply side while considering the transmission and distribution losses. LEAP software is used to construct the Low Carbon Power Model of the GBA model, and the model framework is shown in Fig. 9.

### Total electricity demand calculation method

Electricity demand is mainly influenced by factors such as activity level, energy intensity, and electrification level. Activity levels can be measured by economic indicators (such as industrial value added) or physical indicators (such as miles traveled, passenger turnover, floor space, etc.), and energy intensity can be measured by energy consumption per unit of activity. Total local electricity generation mainly depends on the structure of installed local generation and the generating hours. Purchased electricity is determined by electricity demand and local generation. CO_2_ emissions from the power sector are determined by the power supply structure and the carbon emission factors of each power technology^31^.

Industrial sector electricity demand can be calculated based on the activity level, energy intensity, and electrification level of each branch. It can be expressed as follows:

2 $$E_{P,i} = \sum {AL_{i,j} } \times EI_{i,j} \times EL_{i} + \sum {AL_{k,j} } \times EI_{k,j} \times EL_{k}$$

where *E*_*p, i*_ is the industrial sector electricity demand, *AL*_*i, j*_ is the product output (million ton) of branch *i* product *j* in the industrial sector, *EI*_*i,j*_ is energy intensity of branch *i* product *j* in the industrial sector, expressed in energy consumption per unit of product (million tons of standard coal per ton), *EL*_*i*_ is the electrification level of branch *i*, and *i* is the traditional industries in the industry sector. *AL*_*k,j*_ is the industrial value added (billion yuan) branch *k* product *j* in the industrial sector, *EI*_*k,j*_ is the energy intensity of branch *k* product *j* in the industrial sector, expressed in energy consumption per unit of industrial value added (million tons of standard coal per billion yuan), *EL*_*k*_ is the electrification level of branch *k*, and *k* is the emerging industries in the industry sector.

The building sector is divided into commercial buildings and residential buildings. The commercial building electricity demand can be calculated based on the building area, energy intensity, and electrification level. It can be expressed as follows:

3 $$E_{{P,b_{1} }} = \sum {A_{i} } \times EI_{i} \times EL_{i}$$

where* E*_*P,b1*_ is commercial building electricity demand, *A*_*i*_ is the building area of branch *i* (million square meters), *EI*_*i*_ is unit area energy consumption of branch *i* (tons of standard coal per m^2^·a ), *EL*_*i*_ is electrification level of branch *i*.

The residential building electricity demand is calculated based on per capita living area, population, urbanization rate, energy intensity, and electrification level. It can be expressed as follows:

4 $$E_{{P,b_{2} }} = \sum {A_{j} } \times P_{j} \times UR_{j} \times EI_{UR,j} \times EL_{UR,j} + \sum {A_{j} } \times P_{j} \times (1 - UR_{j} ) \times EI_{R,j} \times EL_{R,j}$$

where *EP,b*_*2*_ is residential building electricity demand, *A*_*j*_ is the per capita living area in administrative area *j* (square meter), *P*_*j*_ is the population in administrative area *j*, *UR*_*j*_ is the urbanization rate in administrative area *j*, *EI*_*UR,j*_ is energy intensity of urban residential life in administrative area *j*, *EL*_*UR,j*_ is electrification level of urban residential life in administrative area* j*, *EI*_*R,j*_ is energy intensity of rural residential life in administrative area *j*, *EL*_*R,j*_ is electrification level of rural residential life in administrative area *j*.

Electricity demand in the transportation sector can be calculated based on the activity level, energy intensity, and electricity consumption per unit activity level of each transportation type^32^. It can be expressed as follows:

5 $$E^{\prime}_{P,t} = \sum {E_{i} } \times L_{i} \times N_{i} \times EL_{i} + \sum {E_{j} } \times L_{j} \times EL_{j}$$

where *E' P,t* is transportation sector electricity demand, *E*_*i*_ is 100 km energy consumption of transport *i* (tons of standard coal per 100 km), *L*_*i*_ is annual mileage of transport *i* (per vehicle-km), *N*_*i*_ is ownership of transport *i* (vehicles), *EL*_*i*_ is the percentage of new energy vehicles in transport *i,* and *i* is the type of urban passenger transport other than the metro, *E*_*j*_ is energy consumption per unit passenger turnover or freight turnover of transport *j* (tons of standard coal per passenger-km) or (tons of standard coal per tonne-km), *L*_*j*_ is activity level of transport *j* (passenger-km) or (tonne-km), *EL*_*j*_ is electrification level of transport *j*. And *j* is the type of transport except* i*.

Electricity demand in the agricultural sector can be calculated by activity level, energy intensity, and electrification level. Considering the data availability, the value added of the primary industry is used as the activity level of the agricultural sector. It can be expressed as follows:

6 $$E_{P,a} = AL \times EI \times EL$$

where *E*_*P,a*_ is agricultural sector electricity demand, *AL* is the activity level of the agricultural sector (billion yuan), *EI* is energy consumption per activity level (million tons of standard coal per billion yuan), and *EL* is the electrification level of the agricultural sector.

The total electricity demand is the sum of Eqs. (1), (2), (3), (4), (5). It can be expressed as follows:

7 $$E_{P,t} = E_{P,i} + E_{{P,b_{1} }} + E_{{P,b_{2} }} + E_{P,t}{\prime} + E_{P,a}$$

In the power supply module, the electrical transformation and distribution include transmission line losses, transformer losses, and other equipment losses. Considering the availability of data, only transmission line losses are considered here. The average line loss rate of 3.63%^3^ in Guangdong Province in 2020 is used as the average transmission line loss share of electric utilities in the GBA in that year. Activity levels, energy intensity, and electrification levels for each sector are derived from statistical yearbooks and research and analysis by the project team^33–37^.

### The specification of generation technologies

The power generation module includes coal-fired power, oil-fired power, gas-fired power, hydro-power, nuclear power, wind power, biomass power, and photovoltaic power. For each generation technology, the dispatch rule, process efficiency, historical production, exogenous capacity, maximum availability, merit order, dispatch-able, lifetime, etc. are shown in Table 4.

**Table 4:** The specification of generation technologies in 2020.

Generation type	Process efficiency (%)	Historical production (100 GWh)	Installed capacity (MW)	Maximum availability (%)	Merit order	Dispatchable	Lifetime (year)
Coal-fired power	36.7	1192.3	29,072.5	62	2	Yes	30
Oil-fired power	40.2	19.8	1231.9	60	2	Yes	30
Gas-fired power	58.0	958.2	31,101.0	90	2	Yes	30
Hydro-power	86.0	57.5	1509.4	40	1	Yes	30
Nuclear power	32.0	669.7	9620.0	86	1	Yes	40
Wind power	20.0	67.1	929.1	23	1	Yes	20
Biomass power	25.0	44.0	1565.9	75	1	Yes	30
Photovoltaic power	20.0	21.1	3318.3	16	1	Yes	25

### Methodology for calculating carbon emissions in the power sector

Carbon emissions from the power sector include carbon emissions from local power generation and purchased power. Carbon emissions from local power generation can be calculated based on the energy consumption structure of local power generation and carbon emission factors. The carbon emission of purchased electricity can be calculated based on the purchased electricity and the average CO_2_ emission factor of Guangdong electricity. It can be expressed as follows:

8 $$E_{{CO_{2} }} = \sum {EG_{i,j} } \times bf_{i,j} \times EF_{i} + PE \times EMF$$

where *ECO*_*2*_ is carbon emissions from the power sector, *EG*_*i,j*_ is electricity generation from category *i* fuel *j* power plant, *bf*_*i,j*_ is standard coal consumption rate for power generation for category *i* fuel *j* power plant, *EF*_*i*_ is CO_2_ emission factor for category *i* fuel, *PE* is purchased electricity, *EMF* is the average CO_2_ emission factor of Guangdong province^38^. The average carbon emission factor of the future power grid in Guangdong Province is determined by the future power generation structure in Guangdong Province.

### Transformation costs methodology

Transformation costs include fuel demand costs, transformation capital costs, and fixed and variable O&M costs^14^. It can be expressed as follows:

9 $$C_{t} = \sum {(c_{i} } \times IC_{i} + f_{i} \times IC_{i} + \upsilon_{i} \times EG_{i} ) + \sum {c_{j} \times D_{j} }$$

where *C*_*t*_ is the total cost, *c*_*i*_ is the capital cost of power technology *i*, *IC*_*i*_ is installed capacity of power technology *i*, *f*_*i*_ is the fixed O&M costs for power technology *i*, *v*_*i*_ is the variable O&M costs for power technology *i*, *EG*_*i*_ is electricity generation by power technology *i*, *c*_*j*_ is usage cost of fuel *j*, *D*_*j*_ is the demand for fuel *j*. The various cost parameters are shown in Table 5^39–41^.

**Table 5:** Electricity production costs in 2020 (unit: billion yuan).

Generation type	Capital cost	Fixed O&M cost	Variable O&M cost	Cost of fuels	Total
Coal-fired power	8.82	11.34	0.12	29.48	49.76
Oil-fired power	0.08	0.20	0	8.25	8.53
Gas-fired power	3.25	5.92	0.10	21.46	30.73
Hydro-power	0.28	0.46	0.01	–	0.75
Nuclear power	4.35	4.89	0.15	0.04	9.43
Biomass power	0.22	0.37	0.01	–	0.60
Photovoltaic power	0.17	0.56	0	–	0.73
Wind power	0.08	0.38	0	–	0.46
Total	17.24	24.12	0.39	59.24	100.99

Previous studies have typically used learning curve models to predict trends in generation costs. The learning curve model based on the “learning by doing” effect can be formulated as follows:

10 $$c_{i} (n) = c_{i} (0) \times IC_{i} (n)^{{ - \alpha_{i} }}$$

11 $$L_{i} = \frac{{c_{i} (0) \times (2IC_{i} (n))^{{ - \alpha_{i} }} }}{{c_{i} (0) \times (IC_{i} (n))^{{ - \alpha_{i} }} }} = 2^{{ - \alpha_{i} }}$$

12 $$LR_{i} = 1 - L_{i} = 1 - 2^{{ - \alpha_{i} }}$$

where *c*_*i*_(*n*) is the generation cost of power generation technology *i* in year *n*, *c*_*i*_(*0*) is the initial cost per unit of power generation technology *i*, *IC*_*i*_(*n*) is the cumulative power generation capacity of power generation technology *i* in year *n*, *α*_*i*_ is the elastic coefficient of power generation technology *i*, *L*_*i*_ is technology learning rate of power generation technology *i*, *LR*_*i*_ is the technological progress rate of power generation technology *i*. Learning rates for wind power, photovoltaic, coal power, CCUS, biomass power, etc. refer to relevant references^42–46^.

### Socioeconomic parameters assumptions

The GDP growth rate, population, urbanization rate, and industrial structure of the GBA in 2020 are statistically calculated concerning the Statistical Yearbook of the nine prefecture-level cities of Guangdong Province within the GBA, the Statistical Yearbook of Hong Kong and the Statistical Yearbook of Macao in 2019–2020. The GDP growth rate of GBA from 2020 to 2035 is set concerning the existing growth rate trend and the *Outline of the Fourteenth Five-Year Plan for the National Economic and Social Development of Guangdong Province and the Visionary Goals for 2035*^47^, and the industrial structure from 2020 to 2035 is set by the trend of changes in the existing industrial structure and the *Outline of the Fourteenth Five-Year Plan for the National Economic and Social Development of Guangdong Province and the Visionary Goals for 2035*^47^, meanwhile, the impact of changes in GDP growth rate and industrial structure under the 2020–2021 epidemic is considered comprehensively. The GDP growth rate and industrial structure in 2035–2060 are set according to the trend of GDP growth rate and industrial structure change in 2020–2035. The population growth rate of GBA in 2020–2030 is set concerning the *Population Development Plan of Guangdong Province* (*2017–2030*)^48^, and the population growth rate in 2031–2060 is set concerning the trend of population growth rate in 2020–2030. The urbanization rate of GBA in 2020–2035 is set concerning the *New Urbanization Plan of Guangdong Province* (*2021–2035*)^49^, and the population growth rate in 2035–2060 is set concerning the trend of change in urbanization rate in 2020–2035. The socioeconomic parameters setting is shown in Table 6. Discount rate analysis and projections based on historical discount rate trends.

**Table 6:** Key assumptions module parameter settings.

Year	GDP growth rate (%)	Population growth rate (%)	Urbanization rate (%)	Industrial structure
2020	4.9	2.4	88.45	1.36:31.66:66.98
2025	4.7	1.8	89.55	1.35:29.98:68.67
2030	4.5	1.2	90.55	1.34:28.32:70.34
2035	4.2	0.6	91.45	1.32:27.40:71.28
2040	3.8	0.1	92.25	1.30:26.53:72.17
2045	3.3	− 0.1	92.95	1.28:26.02:72.70
2050	2.9	− 0.3	93.55	1.27:25.50:73.23
2055	2.6	− 0.5	94.05	1.25:25.08:73.67
2060	2.3	− 0.8	94.45	1.24:24.68:74.08

## Data Availability

In this study, the data on electricity demand, population, and GDP used for prediction are from the website: http://stats.gd.gov.cn/ (accessed on 19 September 2023), and the data on installed power, electricity generation, and energy consumption are derived from a study of individual power plants.

## Abbreviations

LEAP: Low emissions analysis platform
GBA: Guangdong, Hong Kong, and Mocal Greater Bay Area
CCUS: Carbon capture, utilization and storage
BECCS: Bioenergy with carbon capture and storage
STIRPAT: Stochastic impacts by regression on population, affluence, and technology
O&M: Operation and maintenance
CEG: Clean energy generation
NGE: Natural gas generation
BAU: Business-as-usual
PV: Photovoltaic
m^2^·a: M^2^ multiplied by age
